# Author Correction: An atomically controlled insulator-to-metal transition in iridate/manganite heterostructures

**DOI:** 10.1038/s41467-025-55965-0

**Published:** 2025-01-24

**Authors:** Enyang Men, Deyang Li, Haiyang Zhang, Jingxin Chen, Zhihan Qiao, Long Wei, Zhaosheng Wang, Chuanying Xi, Dongsheng Song, Yuhan Li, Hyoungjeen Jeen, Kai Chen, Hong Zhu, Lin Hao

**Affiliations:** 1 Anhui Key Laboratory of Low-Energy Quantum Materials and Devices, High Magnetic Field Laboratory, HFIPS, Chinese Academy of Sciences, Hefei, Anhui China; 2 Science Island Branch of Graduate School, University of Science and Technology of China, Hefei, China; 3 National Synchrotron Radiation Laboratory, University of Science and Technology of China, Hefei, China; 4 Information Materials and Intelligent Sensing Laboratory of Anhui Province, Key Laboratory of Structure and Functional Regulation of Hybrid Materials of Ministry of Education, Institutes of Physical Science and Information Technology, Anhui University, Hefei, China; 5 Department of Physics, Beijing Normal University, Beijing, China; 6 Department of Physics, Pusan National University, Busan, South Korea; 7 Department of Physics, University of Science and Technology of China, Hefei, China

**Keywords:** Surfaces, interfaces and thin films, Phase transitions and critical phenomena, Electronic properties and materials, Magnetic properties and materials

Correction to: *Nature Communications* 10.1038/s41467-024-52616-8, published online 28 September 2024

In the original version of this Article, the Supplementary Information file was incorrectly referred to throughout the text as:

“See Supplemental Material at [URL will be inserted by publisher] for additional information on sample characterization and control experiments).”

This has been replaced by references to the Supplementary Notes.

The reference to the Supplementary Information file in sentence 8 in paragraph 1 of the subsection “Emergent insulator-to-metal transition in CIO/LSMO heterostructures” of the Results and Discussion section now reads:

“see Supplementary Note 2.”

The reference to the Supplementary Information file in sentence 6 in paragraph 2 of the subsection “Emergent insulator-to-metal transition in CIO/LSMO heterostructures” of the Results and Discussion section now reads:

“see Supplementary Note 10.”

The reference to the Supplementary Information file in sentence 7 in paragraph 2 of the subsection “Emergent insulator-to-metal transition in CIO/LSMO heterostructures” of the Results and Discussion section now reads:

“see Supplementary Note 3.”

The reference to the Supplementary Information file in sentence 9 in paragraph 2 of the subsection “Emergent insulator-to-metal transition in CIO/LSMO heterostructures” of the Results and Discussion section now reads:

“see Supplementary Note 10.”

The reference to the Supplementary Information file in sentence 2 in paragraph 3 of the subsection “Emergent insulator-to-metal transition in CIO/LSMO heterostructures” of the Results and Discussion section now reads:

“see Supplementary Note 4.”

The reference to the Supplementary Information file in sentence 5 in paragraph 3 of the subsection “Emergent insulator-to-metal transition in CIO/LSMO heterostructures” of the Results and Discussion section now reads:

“see Supplementary Note 5.”

The reference to the Supplementary Information file in sentence 3 in paragraph 2 of the subsection “Percolation nature of the insulator-to-metal transition” of the Results and Discussion section now reads:

“see Supplementary Note 4.”

The reference to the Supplementary Information file in sentence 8 in paragraph 3 of the subsection “Percolation nature of the insulator-to-metal transition” of the Results and Discussion section now reads:

“see Supplementary Note 7.”

This has now been corrected in both the PDF and HTML versions of the Article.

