# Supplementary material for: An atomically controlled insulator-to-metal transition in iridate/manganite heterostructures
Source: Nat Commun. 2024 Sep 28;15:8427. doi: 10.1038/s41467-024-52616-8 (PMC11439077; doi:10.1038/s41467-024-52616-8)
Supplement: Supplementary file 1 — Supplementary Information [file 41467_2024_52616_MOESM1_ESM.pdf]

Supplementary information

An atomically controlled insulator-to-metal transition in iridate/manganite heterostructures

Enyang Men[1,2], Deyang Li[1,2], Haiyang Zhang[1,2], Jingxin Chen[1,2], Zhihan Qiao[1,2], Long Wei[3], Zhaosheng Wang[1], Chuanying Xi[1], Dongsheng Song[4], Yuhan Li[5], Hyoungjeen Jeen[6], Kai Chen[3,*], Hong Zhu[7,*], Lin Hao[1,*]

[1]Anhui Key Laboratory of Low-Energy Quantum Materials and Devices, High Magnetic Field Laboratory, HFIPS, Chinese Academy of Sciences, Hefei, Anhui 230031, China

[2]Science Island Branch of Graduate School, University of Science and Technology of China, Hefei 230026, China

[3]National Synchrotron Radiation Laboratory, University of Science and Technology of China, Hefei 230026, China

[4]Information Materials and Intelligent Sensing Laboratory of Anhui Province, Key Laboratory of Structure and Functional Regulation of Hybrid Materials of Ministry of Education, Institutes of Physical Science and Information Technology, Anhui University, Hefei 230601, China

[5]Department of Physics, Beijing Normal University, Beijing 100875, China

[6]Department of Physics, Pusan National University, Busan 46241, South Korea

[7]Department of Physics, University of Science and Technology of China, Hefei 230026, China

CONTENTS

Supplementary Note 1. In-house X-ray diffraction measurements 3

Supplementary Note 2. Transmission electron microscope measurements 4

Supplementary Note 3. Atomic/Electrostatic force microscope measurements 6

Supplementary Note 4. Physical properties of LSMO-LSAT film 8

Supplementary Note 5. Saturated magnetizations at the base temperature 9

Supplementary Note 6. X-ray reciprocal space mapping 10

Supplementary Note 7. Summary of transition temperatures 12

Supplementary Note 8. Fitting detail of XAS 13

Supplementary Note 9. Control experiments on titanate/iridate heterostructures 15

Supplementary Note 10. MR of single iridate and manganite films 17

Supplementary Note 11. Schematic illustration of the effective correlation-modulated percolation 19

Supplementary Note 1. In-house X-ray diffraction measurements

As shown in Supplementary Fig. 1, the diffraction peak of LSMO gradually shifts to lower angles, suggesting that the out-of-plane lattice constant increases with the CIO thickness when $n \leq 3$. Meanwhile, when n<10, the CIO peak cannot be seen from the XRD results, as also observed from RSM measurements in Supplementary Fig. 8.

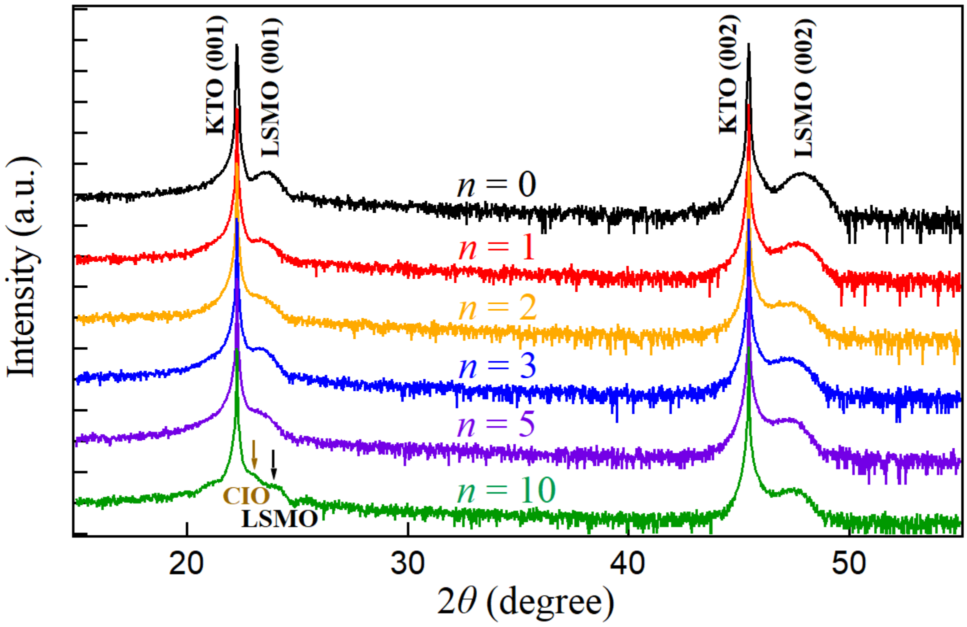

Supplementary Figure 1| θ-2θ patterns of *n*CIO/20LSMO (*n*=0, 1, 2, 3, 5, 10-unit cells) heterostructures.

Supplementary Note 2. Transmission electron microscope measurements

As shown in Supplementary Fig. 2, we present the enlarged view of the CIO-LSMO interface as well as the LSMO-KTO interface on the right panels. It is clear that both interfaces are sharp. The surface of the heterostructure, while is also well identified, is slightly damaged during TEM sample preparation.

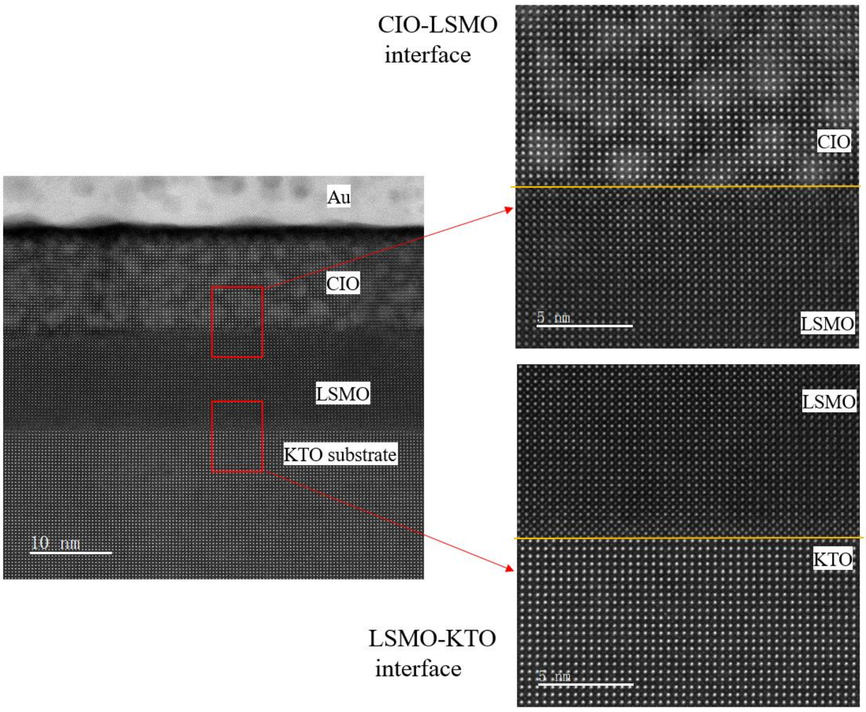

Supplementary Figure 2| A large-area TEM image of the 30CIO/30LSMO heterostructure.

To further verify the high quality of CIO/LSMO interface, we also prepared a 4CIO/18LSMO heterostructure capped with $SrTiO_3$(STO) of 10 u.c.. As shown in Supplementary Fig. 3, we present TEM images of several regions, where both the LSMO/KTO interface and the CIO/LSMO interfaces are clear to be seen. The good quality of interface is in consistent with the observation on the 30CIO/30LSMO heterostructure. This consistency is reasonable because the interface quality should be

independent on n considering the bottom-to-top growth method.

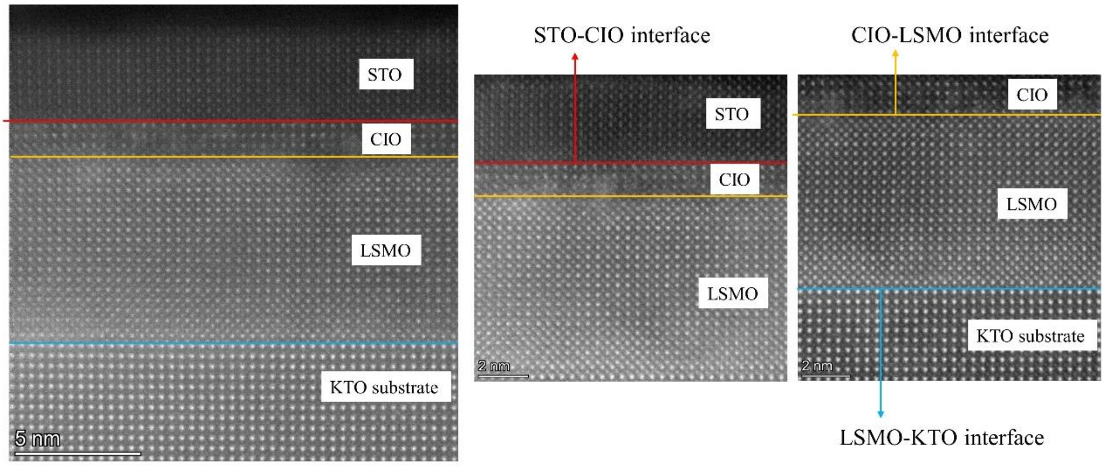

Supplementary Figure 3| Representative TEM images of the 10STO/4CIO/18LSMO heterostructure.

Supplementary Note 3. Atomic/Electrostatic force microscope measurements

The surface topography was characterized by the atomic/electrostatic force microscope technique. Supplementary Fig. 4 shows that the surfaces of all the heterostructures as well as the LSMO-LSAT film are very flat. The surface roughness is summarized in Supplementary Table 1.

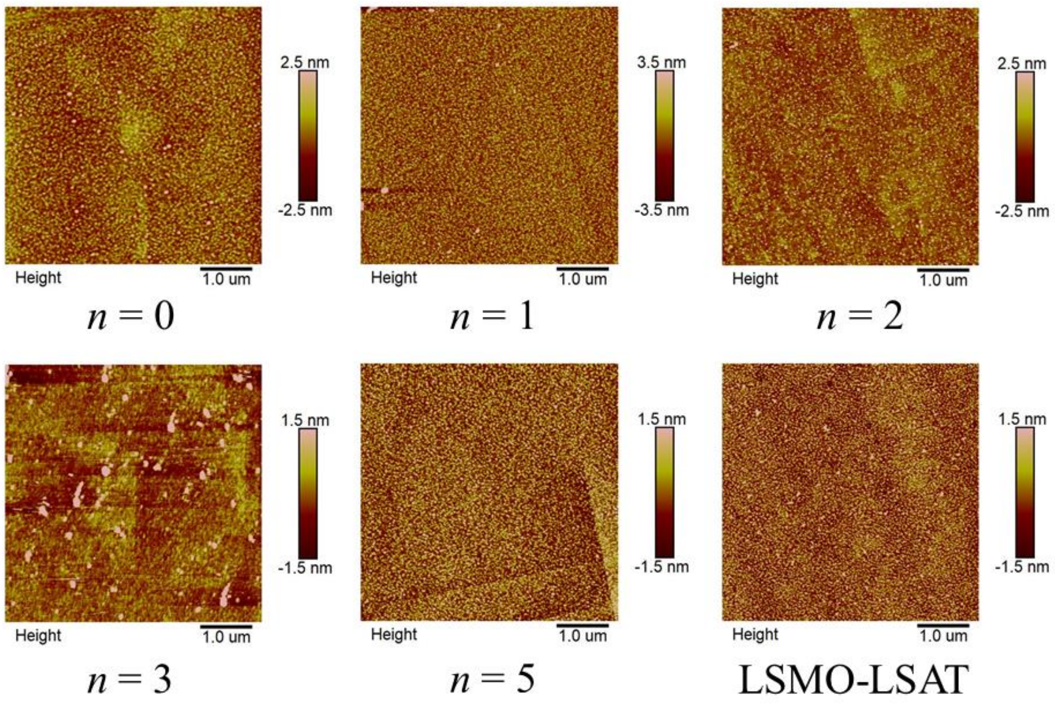

Supplementary Figure 4| Atomic force microscopy images of *n*CIO/20LSMO heterostructures and LSMO-LSAT film.

Supplementary Table 1| The roughnesses of *n*CIO/20LSMO heterostructures and LSMO-LSAT film.

	$n = 0$	$n = 1$	$n = 2$	$n = 3$	$n = 5$	LSMO-LSAT
R_q (nm)	0.639	0.991	0.710	0.765	0.669	0.652
R_a (nm)	0.481	0.722	0.499	0.378	0.517	0.513

Showing in Supplementary Fig. 5 are EFM images of two representative samples. A cluster-like feature with a high contrast was observed on the LSMO film, unveiling a non-uniform electric conductivity on the sample. This is parallel to the scenario of phase separation, where metallic clusters are embedded in an insulating background. On the contrary, the topography is much more uniform in the 3CIO/20LSMO heterostructure, in consistent to the enlarged metallic clusters and the connection of them due to the interfacial effects.

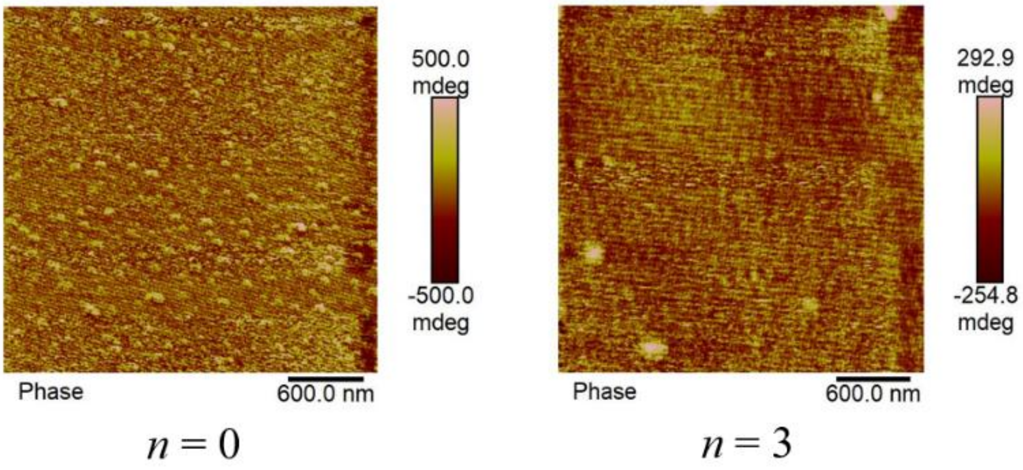

Supplementary Figure 5| Electrostatic force microscopy images of LSMO film (left panel) and 3CIO/20LSMO (right panel) heterostructure.

Supplementary Note 4. Physical properties of LSMO-LSAT film

As shown in Supplementary Fig. 6a, the LSMO-LSAT film displays a profound metallic behavior, in consistent with the double-exchange dominated metallic ground state in a strain-free manganite. Supplementary Figs. 6b&c show the temperature dependence of the magnetization of LSMO-LSAT film measured at $H = 0.1$ T, and the hysteresis loop at 50 K with the magnetic field along the in-plane direction. The onset temperature of the LSMO-LSAT film is about 335K. The coercive field and saturated magnetization are about 20 Oe and 470 emu/cm^3, respectively.

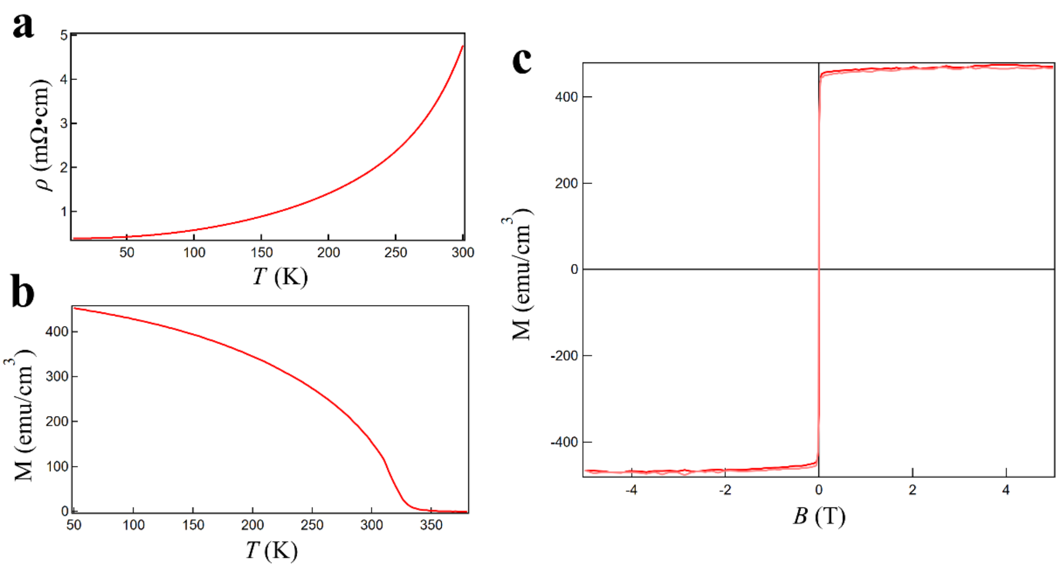

Supplementary Figure 6| (a) ρ-T curve, **(b)** M-T curve, and **(c)** M-H loop of the LSMO-LSAT film.

Supplementary Note 5. Saturated magnetizations at the base temperature

Supplementary Fig. 7 shows the hysteresis loop at 5 K with the magnetic field along the in-plane direction. With the increase of CIO thickness, the saturated magnetization of *n*CIO/20LSMO heterostructures (*M*) increases sharply at first and then ceases to increase when *n* is larger than 3. The maximized magnetization of the heterostructures is about half that in the LSMO-LSAT film. The coercivity fields are summarized in supplementary Table 2.

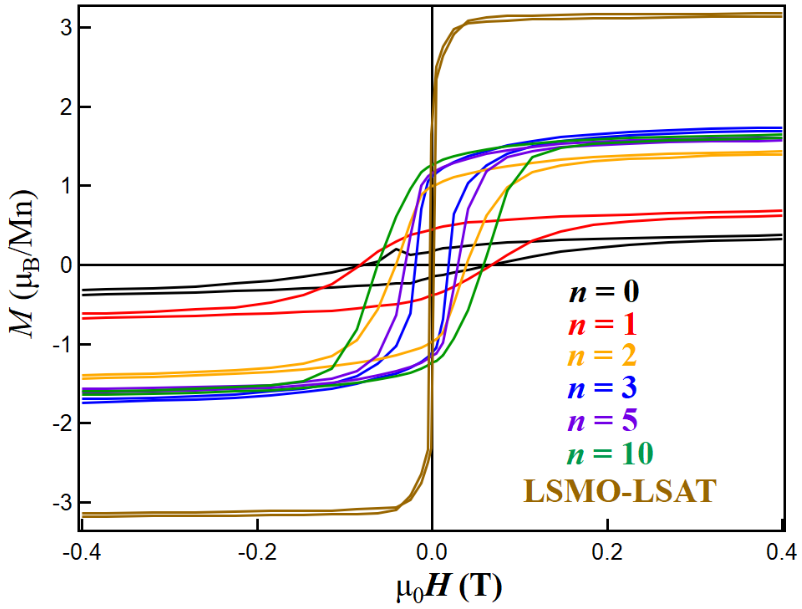

Supplementary Figure 7| Enlarged *M*-*H* loops of *n*CIO/20LSMO heterostructures and LSMO-LSAT film.

Supplementary Table 2| H_c of heterostructures and the LSMO-LSAT film

	$n = 0$	$n = 1$	$n = 2$	$n = 3$	$n = 5$	$n = 10$	LSMO-LSAT
H_c (Oe)	775.3	823.6	417.6	195.2	311.2	620.6	23.1

Supplementary Note 6. X-ray reciprocal space mapping

Supplementary Fig. 8 shows the X-ray reciprocal space mapping measurements of *n*CIO/20LSMO (*n* = 0, 2, 3, 5, 30-unit cells) heterostructures around the (113)-peak of the KTO substrate. For $n \leq 5$, only LSMO block is detected because the CIO blocks are too thin. The CIO block is observable in the 30CIO/20LSMO heterostructure, where the CIO block is relaxed. On the other hand, it is clear that LSMO blocks are fully strained in all the heterostructures

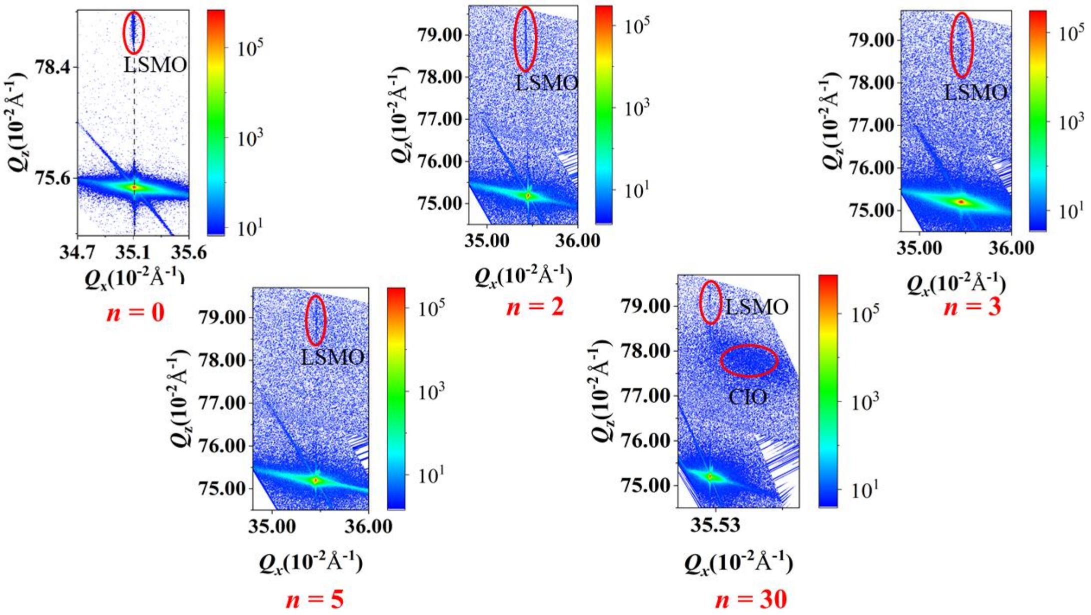

Supplementary Figure 8| RSMs of *n*CIO/20LSMO (*n*=0, 2, 3, 5, 30-unit cells) heterostructures. The peak positions of LSMO and CIO (if presented) are denoted by an oval.

Supplementary Fig. 9 shows the X-ray reciprocal space mapping measurements of the 30LSMO film and 35LSMO film around the (113)-peak of the KTO substrate. The (113) peaks of these films are more extending than that of the 20LSMO film in Supplementary Fig. 8, which indicates that both films are relaxed, and the 35LSMO film is more relaxed than the 30LSMO film.

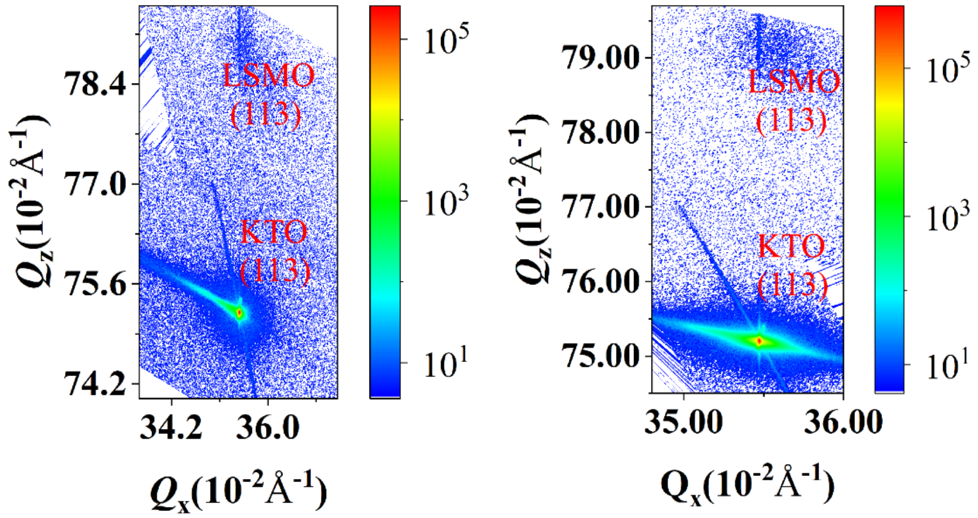

Supplementary Figure 9| RSMs of the 30LSMO film (left) and 35LSMO film (right).

Supplementary Note 7. Summary of transition temperatures

Supplementary Table. 3 summarizes the magnetic ordering temperature determined from magnetic measurements as well as the magnetotransport measurements. T_c is extracted from the deflection point of the *M-T* curves in Fig. 2b, and T_{MR} corresponds to the temperature that maximizes the MR effect in Fig. 2c. T_c is almost the same as T_{MR} in all the heterostructures and LSMO-LSAT films, indicating a double-exchange dominated magnetic ordering in the system.

Supplementary Table 3| T_c and T_{MR} of *n*CIO/20LSMO heterostructures and LSMO-LSAT film.

	$n = 1$	$n = 2$	$n = 3$	$n = 5$	$n = 10$	LSMO-LSAT
T_c	202 K	231 K	260 K	254 K	264 K	312 K
T_{MR}	201 K	227 K	262 K	253 K	266 K	315 K

Supplementary Note 8. Fitting detail of XAS

We employed multiplet calculations for Mn^{3+} and Mn^{4+} to conduct quantitative fittings and determine the mean valence of Mn in CIO/LSMO heterostructures, as illustrated in Supplementary Fig. 10. By utilizing the configuration interaction cluster model and parameters from a previous study, we performed calculations for Mn^{3+} and Mn^{4+} using CTM4XAS. The calculated spectra closely match the experimental results for Mn^{3+} from $LaMnO_3$ and Mn^{4+} from Li_2MnO_3. By combining these two spectra, we were able to quantitatively reproduce our experimental spectra of CIO/LSMO with n = 0 and n = 5 as 50% Mn^{4+} + 50% Mn^{3+} and 40% Mn^{4+} + 60% Mn^{3+}, respectively. The XAS profiles of samples with n = 1, 2, and 3 were nearly identical to that of the n = 5 sample (Fig. 3a). Consequently, we estimated the mean valence of 3.5 and 3.4 for Mn in the n = 0 and n = 1, 2, 3, 5 samples, respectively. This allowed us to determine a charge transfer value of 0.1 e/Mn. The determined valence of $Mn^{3.5+}$ for the LSMO film slightly exceeded the expected 3.33+ from the chemical formula $La_{0.67}Sr_{0.33}MnO_3$. This discrepancy may stem from the covalent Mn-O interactions in the calculated Mn^{4+} and Mn^{3+} spectra. The relative intensity between 640.3 and 642.1 eV, which are the crucial features for Mn^{4+} and Mn^{3+}, was used to assess the fitting quality. The fitting uncertainty is approximately ±0.03 e/Mn.

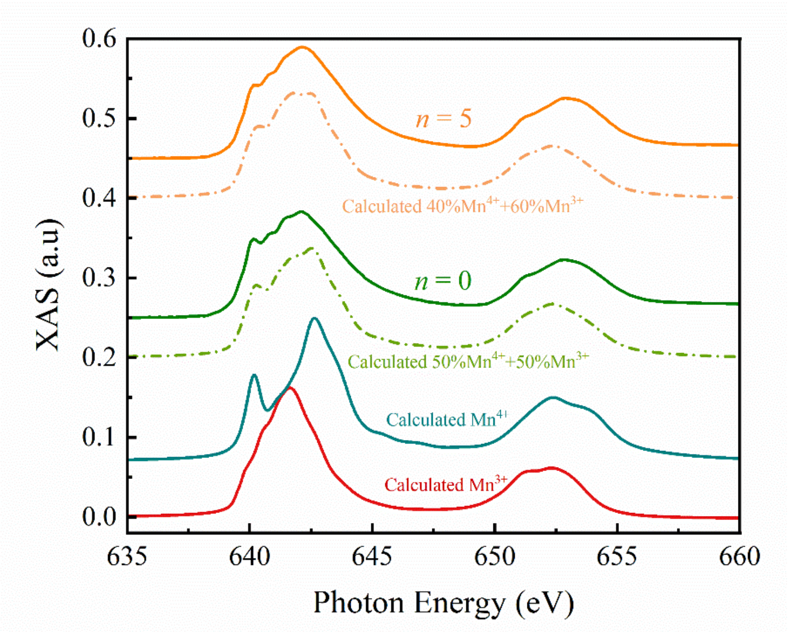

Supplementary Figure 10| The fitting of XAS at Mn *L*-edge with calculated Mn^{4+}, Mn^{3+} spectra using the configuration interaction cluster model.

Supplementary Note 9. Control experiments on titanate/iridate heterostructures

We have prepared $5CaTiO_3$(CTO)/20LSMO and 3CIO/5STO/20LSMO heterostructures in order to further demonstrate the key role of the interfacial effect in the CIO/LSMO heterostructures. As shown in Supplementary Fig. 11, the 5CTO/20LSMO is strongly insulating with the resistivity rapidly increases with decreasing temperature, in stark contrast to the metallic state in 5CIO/20LSMO. From this control experiment, one then can conclude that oxiding effect from Ca is negligible in the observed insulator-to-metal transition. As shown in Supplementary Fig. 12, in contrast the interface-driven lattice expansion in 5CIO/20LSMO, the 5CTO/20LSMO has a smaller out-of-plane lattice parameter. This comparison also indicates that interface effect is weak in the 5CTO/20LSMO heterostructure, in consistent with its robust insulating state.

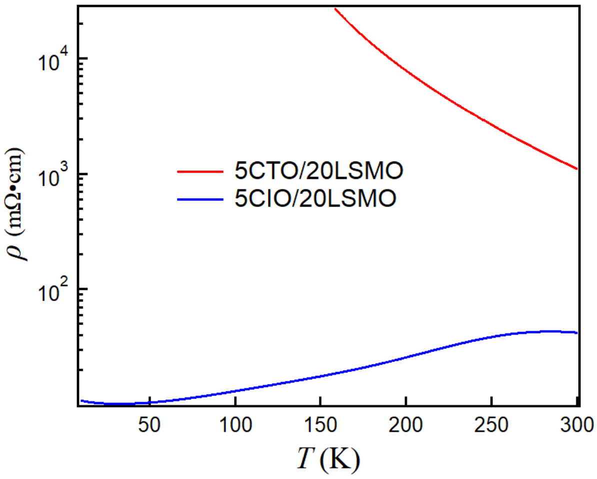

Supplementary Figure 11| The ρ-T curves of 5CTO/20LSMO and 5CIO/20LSMO heterostructures.

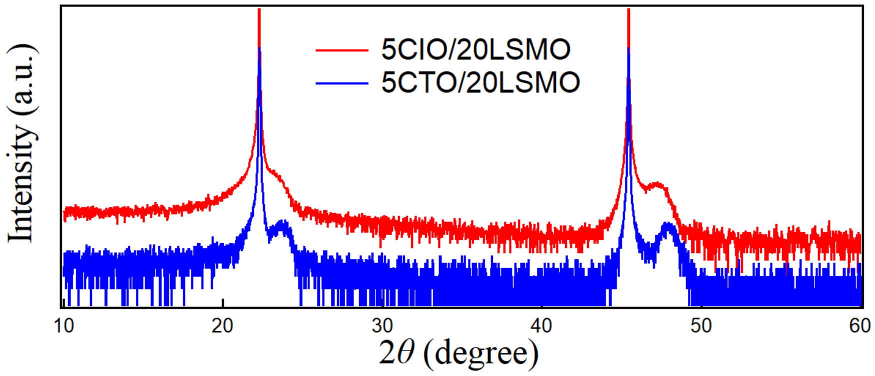

Supplementary Figure 12| The XRD pattern of 5CTO/20LSMO and 5CIO/20LSMO heterostructures.

As shown in Supplementary Fig. 13, in contrast to the metallic behavior of the 3CIO/20LSMO heterostructure, the 3CIO/5STO/20LSMO heterostructure is also insulating. This comparison further confirms that the CIO/LSMO interface plays a dominant role in driving the insulator-to-metal transition.

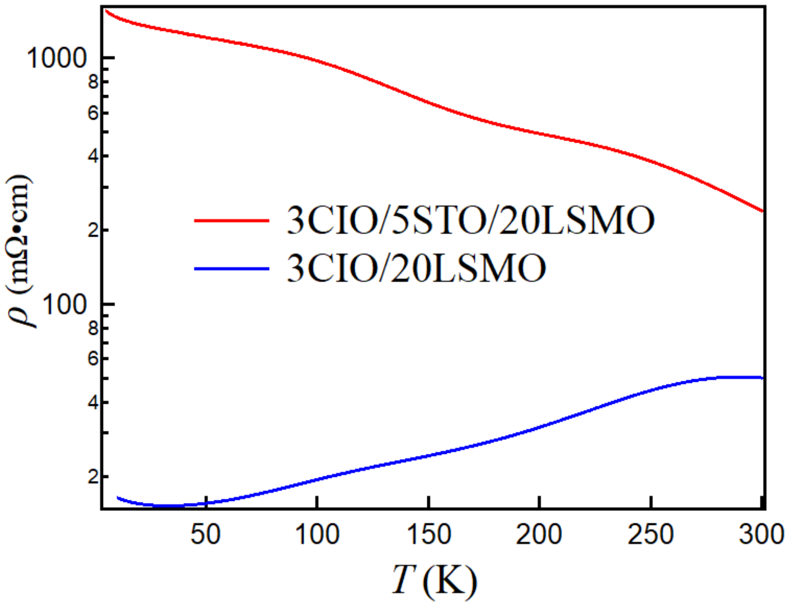

Supplementary Figure 13| The ρ-T curves of 3CIO/5STO/20LSMO and 3CIO/20LSMO heterostructures.

Supplementary Note 10. MR of single iridate and manganite films

We have prepared two CIO films (30 and 50 u.c.). As shown in Supplementary Fig. 14, MR of CIO films is negligible at high temperatures, and the low-temperature MR is about three orders of magnitude smaller than that of the heterostructures. This striking difference allows us to confidently exclude the MR contribution from CIO.

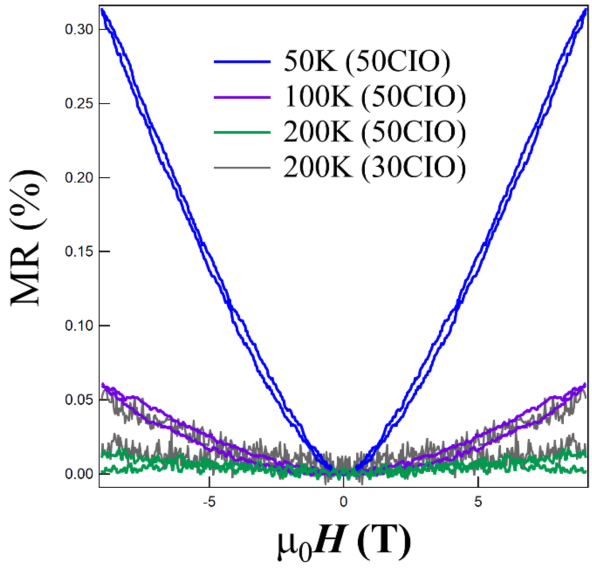

Supplementary Figure 14| MR of 50 u.c. and 30 u.c. CIO films at different temperatures. Note that the 30CIO film is rather insulating, such that a small temperature fluctuation during field scan leads to an artificial hysteresis.

As shown in the Supplementary Fig. 15, MR of the LSMO film is also much smaller than that of the *n*CIO/20LSMO heterostructures.

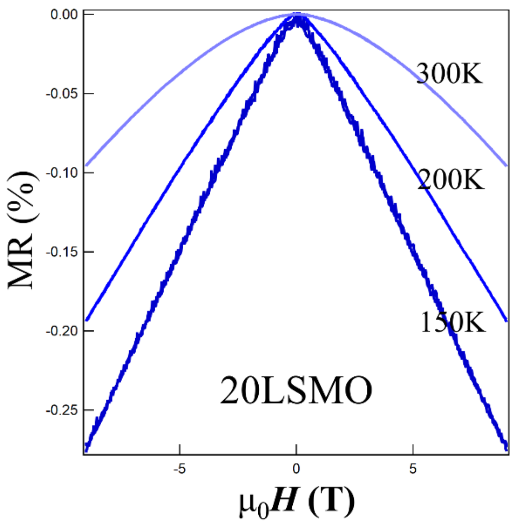

Supplementary Figure 15| MR of the LSMO film at different temperatures.

Supplementary Note 11. Schematic illustration of the effective correlation-modulated percolation

As schematically shown below, the reduced insulating strength of a thick LSMO block, due to the suppressed effective correlation, can be ascribed to the shortened separation between metallic clusters. In this context, it is easier to connect these clusters with the interfacial effect, leading to a complete conducting path in the heterostructure at a smaller *n*. On the other hand, when LSMO is too thin, the metallic clusters are too small to be connected by the interfacial effect, leading to a robust insulating state in the *n*CIO/10LSMO heterostructures.

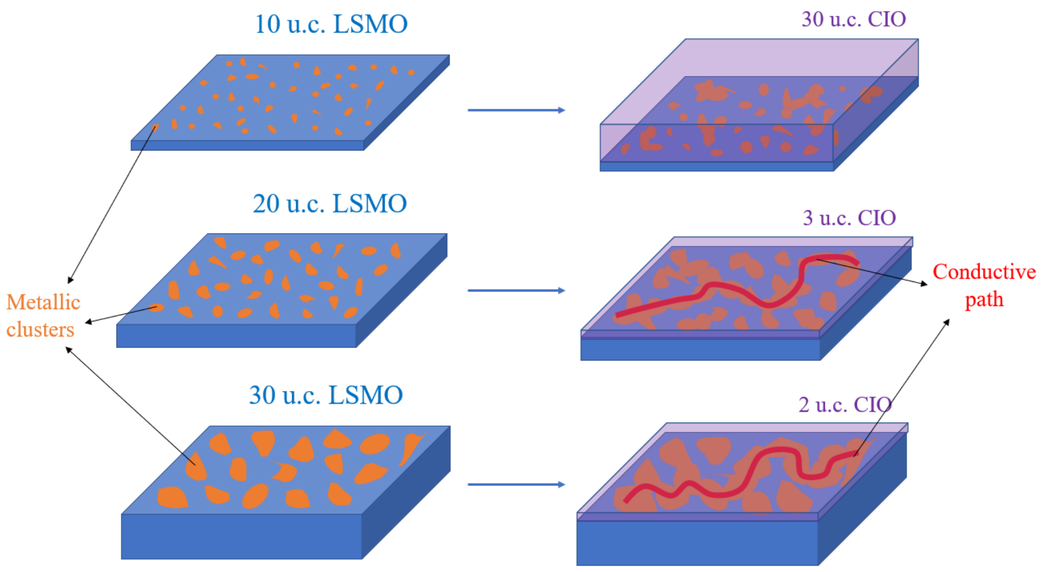

Supplementary Figure 16| The schematical diagram of the percolation-type insulator-to-metal transition. The conductive path is highlighted by red curves.
